# A longitudinal study of the emerging self from 9 months to the age of 4 years

**DOI:** 10.3389/fpsyg.2015.00789

**Published:** 2015-06-10

**Authors:** Susanne Kristen-Antonow, Beate Sodian, Hannah Perst, Maria Licata

**Affiliations:** Department of Psychology, Ludwig-Maximilians-University, Munich, Germany

**Keywords:** longitudinal studies, self concept, social cognition, conceptual development, infancy research

## Abstract

The aim of this study was to investigate if children’s early responsiveness toward social partners is developmentally related to their growing concept of self, as reflected in their mirror self-recognition (MSR) and delayed self-recognition (DSR). Thus, a longitudinal study assessed infants’ responsiveness (e.g., smiling, gaze) toward social partners during the still-face (SF) task and a social imitation game and related it to their emerging MSR and DSR. Thereby, children were tested at regular time points from 9 months to 4 years of age. Results revealed significant predictive relations between children’s responsiveness toward a social partner in the SF task at 9 months and their MSR at 24 months. Further, interindividual differences in children’s awareness of and responsiveness toward being imitated in a social imitation game at 12 months proved to be the strongest predictor of children’s DSR at 4 years, while some additional variance was explained by MSR at 24 months and verbal intelligence. Overall, findings suggest a developmental link between children’s early awareness of and responsiveness toward the social world and their later ability to form a concept of self.

## Introduction

### Self Development: The Importance of Longitudinal Data

The ability to represent oneself as an intentional agent is foundational for the development of social cognition. Meltzoff (1990) has argued that, from birth, infants are able to recognize others as “like me.” Based on this fundamental human ability to establish correspondence between oneself and another agent, infants’ increasing ability to represent themselves as intentional agents leads to an understanding of others’ intentional action. Evidence for this view comes from a study of goal-encoding in very young infants which showed that infants as young as 3 months can encode others’ reaching and grasping actions as goal-directed after having experienced themselves as goal-directed agents with the help of Velcro mittens (Sommerville et al., 2005). While a representation of the self as an intentional agent remains implicit in social interactions throughout the first and second years of life, first evidence for an explicit self-representation emerges close to the second birthday, when children recognize themselves in the mirror and begin to use self-referential language. Theories of the developing self (e.g., Damon and Hart, 1982; Meltzoff, 1990; Rochat, 2003) have emphasized the importance of experience in reciprocal social interaction during the first and second years of life in leading up to the developmental milestone of mirror self-recognition (MSR). Furthermore, MSR has been theoretically linked to later Theory of Mind development (Gallup and Suarez, 1986; Parker et al., 1994). However, to date, there is little evidence for these views, for lack of longitudinal data. If social responsiveness in specific types of early social interactions which provoke self-awareness is developmentally linked to later explicit self-representation, then individual differences in these tasks should be correlated independently of more general cognitive abilities. There is longitudinal evidence for long-term conceptual continuity in understanding *others*’ intentional states from infancy to preschool age (cf. Aschersleben et al., 2008; Wellman et al., 2008; Thoermer et al., 2012), but no comparable studies have been conducted with respect to the self. In the present longitudinal study of self-development, we investigate the developmental relation of two markers of self-representation, indexing different levels of self-awareness: The MSR task at 24 months and the delayed self-recognition (DSR) task at 4 years of age. Note that these classic, yet controversial, tests have been widely used as markers of the self in empirical studies across cultures (e.g., Keller et al., 2005; Broesch et al., 2010), in typical and atypical development (e.g., Povinelli et al., 1996; Lind and Bowler, 2009), as well as across species (e.g., Povinelli et al., 1993). Specifically, we explore the predictive relations between precursor abilities emerging in social interaction in the first and second years of life and these two markers of self-representation.

### Levels of Self-development

The developmental process of self-understanding has been described as “forward engineering” (Rochat, 2003, p. 117, p. 10), meaning that different constituents of the self develop chronologically during infancy and early childhood (Damon and Hart, 1982). This view implies that interindividual differences in competencies at the different theorized levels are related and that the self develops as a differentiated, yet conceptually coherent concept. Rochat described five levels of self-awareness of which two levels are of central interest, since they pertain to MSR and DSR, respectively. The first level of interest is the “identification”-level at which toddlers are able to express an identified self and comprehend that the mirror reflects their self-experienced “me,” not some other individual. In other words, at this level the toddler is able to detect the correspondence between a mental representation of the self and an observed marked mirror image and at the same time is able to differentiate between the two. The second level of interest is the level at which the self can be truly represented independently of featural information and temporal contingency. At this level pre-schoolers begin to recognize themselves in videos and photographs taken in the past as opposed to live videos or contingent mirror images. It is called the “permanence”-level. It has been controversially discussed to what extent toddlers’ MSR and their ability to recognize themselves in videos index self-awareness or a permanent awareness of self, respectively. The following paragraph will discuss different theoretical viewpoints.

### Interpretations of MSR and DSR

Classic interpretations see MSR as an evidence of children’s knowledge about what they look like (Amsterdam, 1972; Lewis and Brooks-Gunn, 1979; Bischof-Köhler, 1988, 1991), since the child is required to use a mirror to detect a mark covertly placed on her or his nose or cheek. In order to do so, the child has to detect the discrepancy between the mental representation of his or her own body (e.g., cheek without rouge) and the observed marked mirror image (e.g., cheek with rouge). Therefore, fitting with Rochat’s (2003) label, children’s mastery of this task at around 18–24 months of age (Amsterdam, 1972; Lewis and Brooks-Gunn, 1979; Asendorpf and Baudonniére, 1993; Nielsen and Dissanayake, 2004) has been regarded as evidence for being able to identify oneself.

This view has been challenged, however. Overall, there are lean interpretations, rich interpretations and proposals somewhere between lean and rich. While some theorists (Lewis and Brooks-Gunn, 1979; Courage and Howe, 2002; Nielsen et al., 2003) state that mastery of this task proves that children know what they look like, leaner interpretations have stated that children pass this test because of kinaesthetic-visual matching skills (Mitchell, 1993; Heyes, 1994). In contrast, richer theories have claimed that beyond identifying themselves, children’s mark-directed behavior is evidential of their underlying introspective abilities and reflective capacities (Gallup, 1998; Gallup et al., 2002). For instance, Bischof-Köhler (1991) argued that the ability for mental imagination is necessary for MSR. Mental imagination involves self-objectification and describes the ability to represent objects, including the self, independently of the immediate perceptual reality (see also Moore et al., 2007). In the case of MSR, the child must be able to couple the “I” (the subject of one’s own experience) with the objectified and reflected-on “Me.” Thereby, the “I” can recognize the mirror image as “Me” (Bischof-Köhler, 1991, p.12). Lewis (2003) considers MSR as an indicator of self-metarepresentation abilities: by recognizing oneself in the mirror, the mental state of “Me” (as opposed to an implicit knowledge of the self) is established. The mental state of “Me” in turn gives rise to mental state attributions to others and awareness of the relation between self and other (Lewis, 2003). In contrast, in a more cautious proposal, Perner (1991) argued that self-recognizers have formed multiple representations of one situation or event, so called “secondary representations” and thus, understand the relation between the real situation and the situation in the mirror. However, MSR does not require a representation of the representational relation between oneself and the mirror image of oneself. Similarly, Suddendorf and Butler (2013) argue that MSR requires the ability to collate representations, rather than a metarepresentational understanding of the relation between these representations. Some proposals have been more domain-specific and state that children’s developing cognitive skills in regard to analyzing their own face result in mastery of the MSR task (e.g., Neisser, 1995). It is argued that it is only when children understand that their face is important to other people in order to identify them, they start to use mirrors to see their reflection.

Empirical evidence seems to rule out extremely lean interpretations of MSR, as well as proposals focusing exclusively on children’s developing recognition of their own face. For instance, Nielsen et al. (2006) showed that children can recognize features of their whole body such as their legs, instead of just their face, in the mirror. Further, when altering children’s appearance by putting them in pants, 24-month-olds updated their representation of what they looked like during an exposure phase that had just lasted for 30 s. Thus, instead of facing problems because their proprioceptive matching capacities were handicapped by wearing pants, they detected the mark more rapidly. They only faced problems when they had not been previously exposed to their altered looks and thus, could not build an expectation of what they were supposed to look like. This result supports that children recognize the mark because of a match between the image in the mirror and their expected image of themselves (see also Moore, 2007).

While MSR measures a temporally restricted self-representation, a representation of oneself as a temporally extended individual seems to develop only around the age of 4 years (e.g., Povinelli et al., 1996). This is what Rochat (2003) calls the “permanent self” and what according to Povinelli (1995) is the “proper self,” characterized by children’s comprehension of the fact that different temporal representations (past, present, future) of the self belong to one underlying unifying entity. The standard task to measure this more elaborate concept of self is the DSR task (Povinelli et al., 1996). In the DSR task children have to relate their current self to their temporally-delayed self as shown on a videotape. Mastery of this task emerges between 3 and 5 years of age (depending on the time-delay between recording and showing the video to the child; cf. Povinelli, 2001). While replicating the developmental asynchrony between MSR and DSR, a study by Suddendorf (1999) has challenged the view that young children have specific problems with self-awareness. Rather, they seem to have general problems in relating information shown in a video to the current situation, whether self-related or not (as in the case of a surprising object in the room). Similarly, in case of the MSR-task, live video versions lead to a significant drop in children’s performance below chance level (Vyt, 2001; Suddendorf et al., 2007). While it remains debatable if these difficulties merely reflect children’s problems with the video as a medium itself (Suddendorf, 1999, 2003; Troseth, 2003), the validity of the DSR task as a measure of self-representation has been questioned. Within-subject longitudinal data on different measures of self-awareness can contribute to a better understanding of this marker.

### The Social Origins of Self

The general idea that social experiences in the first year of life support self-development (e.g., Damon and Hart, 1982; Rochat, 2009) is corroborated by empirical evidence from cross-cultural studies showing that variations in social experiences impact children’s performance at the MSR-task. In cultures with a distal parenting style with a lot of face-to-face contact and object manipulation (e.g., Greece) children recognize themselves earlier than in cultures with a stronger emphasis on body contact and body stimulation (e.g., Cameroon). Cultures (e.g., Costa Rica) utilizing proximal as well as distal parenting practices fall between the two other cultural groups in regard to the onset of children’s MSR (Keller et al., 2004). Further, a parenting style where mothers react more contingently (e.g., German mothers when compared to Nso mothers in Cameroon) has been shown to lead to a higher rate of MSR (Keller et al., 2005).

Thus, contingency detection in reciprocal social interaction appears to be one mechanism supporting the development of self-awareness. Rochat (2009) emphasizes that infants’ engagement in reciprocal social interaction allows them to use the adult as a screen providing an opportunity for self-objectification leading to an emerging sense of shared experience with others. Within these reciprocal exchanges infants can develop a sense of contingency and agency as they experience causal efficacy between their own and the other person’s behavior, as well as bodily reactions. A frequently investigated phenomenon assessing infants’ sensitivity to what is reflected back by their interaction partner is the so-called still-face (SF) effect (Tronick et al., 1978). In reacting with irritation when their interaction partner interrupts the interaction (frozen face), and by showing different gaze and smile patterns while trying to bring one’s social partner back into interaction, as well as by displaying reengagement behaviors such as vocalizing or bodily movements, children show social responsiveness (see Mesman et al., 2009, for a meta-analysis of 39 studies employing the SF task). Individual differences in social responsiveness to contingency disruption may thus be predictive of the age of mastery of MSR and DSR.

Another mechanism promoting self-awareness may be synchronic imitation. Based on infants’ early imitation skills as a “foundation and earliest manifestation” (Meltzoff, 1990, p.141) of the self, infants have ample opportunity to detect the structural equivalence between the acts they perform themselves and the acts they see others perform in everyday interaction. A study by Asendorpf and Baudonniére (1993) found that the extent to which 19-month-old infants engaged in synchronic imitation as a measure of other-awareness was affected by their MSR status. Consistently, strong relations were found between 18-month-olds’ MSR skills and their concurrent imitation skills (Zmyj et al., 2013).This has been interpreted as evidence for a developmental synchrony between self- and other-awareness during imitation. However, a study by Nielsen and Dissanayake (2004) found that while both abilities emerge around the same age, synchronic imitation skills (Asendorpf et al., 1996) and MSR proved to be unrelated. The inconsistent findings may be due to the fact that imitation tasks pose many different cognitive and motivational demands beyond a basic self and other-awareness. A clearer measure of self-awareness can be attained in tasks which tap infants’ awareness of their own actions being mirrored by another person. Meltzoff (1990) designed a social mirroring task, in which 9-to 18-month olds’ had to discriminate between an experimenter mimicking their actions on an identical object and the object “mimicking” them without being manipulated by an experimenter, and found clear evidence for an awareness of being imitated in the majority of the infants above the age of 14 months. Agnetta and Rochat (2004) did not find significant predictive correlations between 14-month-olds’ awareness of being mirrored and their MSR at 18 months. However, the relation was only explored at one measurement point, and only with respect to MSR. If the awareness of being imitated is closely linked to an understanding of others as intentional agents in infants above the age of 12 months as Agnetta and Rochat suggest, then individual differences in this ability may very well be predictive of later DSR which has been theoretically linked to metarepresentation and mental state understanding.

### Hypotheses

Summing up, based on theories distinguishing between different levels of self-development in the sense of one underlying unitary concept (e.g., Rochat, 2003) we assume MSR-skills to be related to DSR-skills. Further, based on Rochat’s (2009) social construction theory and Meltzoff’s (1990) “like me” hypothesis, we assume interindividual differences in (a) social responsivity to contingency disruption, and (b) self-awareness in social mirroring to be predictively related to interindividual differences in a time-restricted concept of self as indexed by MSR-skills, as well as in a temporally-extended concept of self as indexed by DSR-skills. We expect these developmental pathways to be specific and thus, independent of general cognitive abilities.

To test these hypotheses, we studied the predictive relations between interindividual differences (1) in social responsiveness in dyadic interaction (SF reaction) at 9 months of age, (2) in social mirroring assessed at 12 months of age, (3) in MSR at 24 months of age and (4) in DSR at 50 months of age, in a longitudinal design. Interindividual differences based on gender and verbal IQ were systematically taken into account.

## Materials and Measures

### Participants

Overall, 89 full-term children (41 female, 48 male) participated in this comprehensive longitudinal study, while due to attendance the *n* at the later measurement points could vary. The mean age at the first measurement point relevant for this study was 9.00 months (SD = 9 days; *n* = 88), 12.01 months (SD = 7 days, *n* = 89) at the second measurement point and 24.03 months (SD = 8 days; *n* = 81) at the third measurement point. At the fourth measurement point, children were, on average, 4.21 years of age (SD = 0.93 months; *n* = 70). Children were tested in a child-friendly research laboratory at the University of Munich and all came from lower to upper middle-class families in an urban area in the South of Germany. On average, children had one sibling, while the number of siblings ranged from 0 to 3. The study followed the ethical standards for experiments involving humans and was approved of by the University of Munich’s ethics committee.

### Still-face Task

The paradigm was adapted from Striano and Stahl (2005) and involved two interruptive situations (adopting a neutral face and ignoring the child). The main purpose of this task was to measure children’s social responsiveness when confronted with an interruption of communication. Infants were seated on a highchair facing the experimenter at a distance of about 45 cm. Once infants were seated, two identical plastic objects (10 cm of height) were unobtrusively placed to the infant’s left and right side at a distance of about 70 cm. The procedure always started off and finished with a normal interaction (NI), while in between the NI and the two different SF phases were alternated in a randomized order. The five phases lasted for 30 s each. In the NI phase children were involved in a natural dyadic interaction. To render the communication situation as natural as possible, experimenters were free to react intuitively to children’s social interaction bids by talking, singing or laughing. In the SF face-to-face condition, the experimenter adopted a neutral facial expression and looked at the infant’s face without any affect. During the SF ignore phase, the experimenter ensured that the infant held eye contact, then adopted a neutral facial expression and turned to the side of one of the two objects (the side of the objects was counterbalanced across children). Thus, the infant was ignored during the whole phase. No smiling or gazing back at the infant or touching the infant occurred. Thus, the two SF variants did not differ in the facial expression (neutral) and were both characterized by the absence of communicative bids.

Based on the coding scheme by Striano and Stahl (2005), infants’ behaviors were coded using the INTERACT^®^ software. Percent duration of time that infants engaged in a particular behavior was used as dependent measure. The dependent measures included the amount of smiling (raised cheeks, upward turned lips), gazes at experimenter and reengagement behaviors. Reengagement behaviors involved movements (arm or leg movements or pick-me-up gestures accompanied by looks directed at the experimenter) and communicating (e.g., babbling, squeaking, laughing or whining) while gazing at the experimenter. In order to analyze whether a SF effect was manifested, smiling, gaze and reengagement behaviors were averaged across the three NI episodes and then compared to the average duration of smiling, gaze and reengaging behavior across the two SF episodes.

Note that according to the literature competent children should, on average, show important differences in their gaze, smile and behavior when comparing the SF phase with the NI phase. More specifically, the SF effect involves a decrease in smiling and gazing behavior, as well as an increase of reengagement behaviors displayed toward the interaction partner during SF (interrupted) compared to NI episodes.

Thus, for subsequent correlational analyses, difference scores were computed for all three behaviors: the duration of each behavior during NI was subtracted by the duration during SF phases. Based on these difference scores, the following competence levels were defined: for smiling and gazing, a subject was classified as competent (and assigned a competence score of 1) if the difference scores in smiling and gazing behavior between the averaged NI and SF episodes was greater than zero, that is, the child spent less time smiling or gazing respectively, during SF phases than during NI. A child who received a negative value or a score of zero was classified as incompetent (and was assigned a competence score of 0). For reengagement behavior the rationale was different. Note that responsivity to social interaction cues is characterized by an increase of reengagement behaviors after the interruption of an ongoing interaction and a decrease of such behaviors once NI was re-established. Therefore, a competence score of 1 was assigned if the amount of reengagement behaviors during the SF phases was greater than during NI. Note that all scores around 0 and below were assigned a 0. In order to be assigned a score of 1 instead of 0, the differences between the NI and the SF effect had to be significantly different from zero.

Consequently, infants were classified as incompetent (and assigned a competence score of 0) if they did not show an increase of reengagement behaviors during SF episodes. These dichotomous variables were included in correlational analyses within the SF task as well as in correlations with measures from other tasks. A second independent coder, who was blind to the experimental hypotheses, coded a random 30% of all infants and measures for reliability. Cohen’s Kappa for all measures was 0.74. In regard to excluded children, the task was not administered to *n* = 8 children, while *n* = 8 children had to be excluded due to technical errors of the camera system and *n* = 6 children due to crankiness and being fuzzy. An additional five children could not be included in the smiling analyses because they did not display any smiling behavior.

### Social Mirroring Task

This task was adapted from Meltzoff (1990). The main purpose of this task was to test infants’ beginning self-awareness by assessing if they show preference for an experimenter who mirrors their own actions.

Two experimenters sat across the child at a table, while the child was seated within the caretaker’s lap (120 cm away from both experimenters). To make sure children would not develop a preference for one of the experimenters, both experimenters were interacting with the child for about 5–10 min before the experiment started. At the beginning of the experiment, identical toys were handed to both experimenters, as well as to the child at the start of each trial. Each trial lasted for about 45 s. The toys were a car (10 cm long × 9 cm tall), a cup (6.5 cm diameter × 8.5 cm tall), a shovel (20 cm long × 6 cm wide) and a round form (12 cm diameter × 4 cm tall). While the starting object was counterbalanced across infants, in the following, the object order remained the same: the car was always preceded by the cup, the shovel was always preceded by the car, and the form would always follow the shovel independent of the respective starting object. Note that this was done in order to avoid order effects in this comprehensive longitudinal study. We predefined children’s actions. The experimenter to the left always imitated the infant’s actions. Correspondingly, the experimenter to the right performed control actions. The predefined actions (control actions in brackets) were as follows: Shake (Slide), Slide (Shake), Pound (Poke), Poke (Pound), Mouth (Touch Body), Touch Body (Mouth), Passive (Passive). It was made sure that experimenters showed the same activity level, which according to Meltzoff (1990) rules out that the infant prefers one of the experimenters because of the way he or she manipulated the toy. Further, since children might prefer the experimenter who acts temporally contingent upon their own actions, both the imitating and the non-imitating experimenter started and stopped acting at the same time as the infant. The task was filmed and there were three target behaviors which were then coded using the INTERACT^®^ software. The average duration of smiling and looking at either the imitating or the non-imitating experimenter, as well as the number of instances children showed testing behavior averaged across the four objects were used as dependent variables. Thereby, in order to be coded as smiling infants had to display raised cheeks and lips which were turned upward. Testing behavior was defined as sudden and unexpected actions (sudden stop and restart) on the toy while eyeing the experimenter.

To build competence scores, first difference scores were calculated by calculating the duration or number of times (in the case of testing behavior) of a particular behavior which was directed at the imitating experimenter minus the duration or number of times the same behavior was directed at the non-imitating experimenter. First, preferences score were created: Any subject receiving a difference score larger than zero preferred the imitating experimenter and therefore obtained the preference value 1, while subjects who did not differentiate between both experimenters obtained value 0. Subjects preferring experimenter 2 displayed more behavior toward experimenter 2 resulting in a negative difference score. Those infants obtained the preference value –1. Additionally, competence scores were established by merging the preference value –1 and 0. Thus, if infants preferred the non-imitating experimenter 2 or showed no preference for either experimenter they were classified as incompetent and were assigned a score of 0. In contrast, if infants preferred the imitating experimenter they were classified as competent (score of 1). The sum score of the competence scores in regard to all three behaviors was used in analyses. Cohen’s Kappa for the competence scores of all three behaviors ranged from 0.73 to 1.0.

In regard to excluded children, the task was not administered to *n* = 6 infants due to time restrictions, while *n* = 6 infants had to be excluded due to not showing sufficient interest in the objects and *n* = 8 children due to technical problems with the camera system.

### Mirror Self-recognition Task

The task was adapted from Asendorpf and Baudonniére (1993). The main purpose of this task was to assess children’s growing concept of self by testing if children recognize themselves in the mirror.

Prior to testing, child and experimenter engaged in a warm-up phase involving a mirror (dimensions: 110.5 × 104 cm), during which the experimenter made sure the child fixated the mirror for a minimum of three times and at least once for 2 s (baseline-phase). Then, the parent approached the child to apply a mark on either the child’s nose or cheek. This was done in a way to ensure that the parent was not visible in the mirror for the child or that the child and parent were not standing in front of the mirror. The parent used a cloth with lipstick traces (invisible to the child) and wiped the child’s nose thereby leaving a mark on the child’s nose or cheek. For none of the children this served as a clue leading them to touch their face after the mark-application. This application-phase was followed by the test-phase during which the experimenter made sure to focus the child’s attention back on the mirror (e.g., by moving a puppet between the child and the mirror) so that the child would look into the mirror at least three times and at least once for 2 s.

The task was filmed and the videos were analyzed using a coding scheme adopted from the Mirror Behavior Checklist by Amsterdam (1972) in both the baseline-phase and test-phase. A child was coded as 1 (recognizer) if he or she touched the mark [e.g., while verbally referring to either the mark, both the mark and the self or to self (child’s name or I)]. If the mark-touching behavior was not present, the child received a score of 0 (non-recognizer). Interrater-reliability for the recognizer/non-recognizer coding was assessed by calculating Cohen’s Kappa and was 0.92.

In regard to excluded children, the task was not administered in *n* = 3 children due to time restrictions, while *n* = 5 children had to be excluded because could not be brought to focus on the mirror; *n* = 2 did not concentrate during the task and were distracted and *n* = 2 additional infants had to be excluded because of technical problems with the camera system.

### Delayed Self-recognition Task

This task was adapted from Povinelli et al. (1996). The main purpose was to assess children’s understanding of the self as possessing explicit temporal features. At the beginning of the experiment the child and experimenter 1 sat across from each other at a table surrounded by black walls to secure a high contrast-video. Experimenter 2 sat at the child’s right to operate a hand camera which stood ca. Two meters away from the child. Further, a covered video monitor within the child’s visual field, but not yet visible to the child, was part of the setting. The monitor had a width of 39.5 cm and a height of 35 cm. Two cameras were used to film the whole setting, as well as close-ups of the child during the experiment.

At the beginning of the marking-phase, experimenter 2 showed the camera to the child. The child was told that the child and experimenter 1 would play a game and that the camera would record everything so that they could look at the video later. Then, experimenter 1 and the child began playing a search game lasting for five trials, where the child had to search for a cracker which could be hidden under three different opaque containers. Trials 1 and 2 were used to habituate the child to experimenter 2 touching his or her forehead (this was done while praising the child for his or her success at the search task). During trial 3 a sticker (a yellow one was used for dark-haired children and a blue one was used for blond-haired children) was placed at the child’s forehead. The post-it stickers measured 76 × 76 mm. Trials 4 and 5 served as control trials to ensure the child had not detected the sticker. In these trials the child was only praised, but not touched.

The test-phase followed 2 min after trial 5 and experimenter 2 informed the child that they would now look at the video. It was made sure that the overall setting remained the same in the test-phase as compared to the marking-phase and that the child’s face was not reflected in the monitor. The child was shown the video beginning in trial 3 and 15 s after the start the first prompt was given: “Who is this?,” while pointing at the child in the video. If children gave no answer the prompt was repeated. After an additional 15 s, experimenter 2 gave the second prompt and asked: “What is this?,” while pointing at the sticker in the video. If the child did not answer or answered incorrectly the experimenter said: “This is a sticker!” followed by “Where is the sticker now?” After 15 additional seconds the third prompt followed and the experimenter said: “Can you find the sticker? Where it is now?” Thereby, the word “now” was emphasized.

It was coded whether children took off the sticker or touched it, as well as when this behavior occurred. Based on this, children could receive scores ranging from 0 to 4. They received a score of 0 if they did not touch the sticker at all. They received a score of 1 if they touched it only after the third cue, a score of 2 if they touched it after the second cue, a score of 3 if they touched it after the first cue and a score of 4 if they touched it even before any cues were given. Interrater-reliability for this coding was 0.79 (Cohen’s Kappa).

In regard to excluded children, the task was not administered in *n* = 3 children due to time restrictions, while *n* = 9 children detected the sticker during application and in *n* = 2 children the sticker fell off during the play-back phase. Finally, *n* = 1 child did not pay attention to the video at the critical time points.

### WPPSI Verbal IQ Subtest

WPPSI verbal IQ subtest (Petermann, 2009). In order to control for children’s general cognitive skills, at the fourth measurement point, their verbal IQ was assessed and used as a control variable.

For this purpose two subtests of the German version of the WPPSI (Petermann, 2009) verbal IQ scale were administered. The procedure followed the test manual. While the subtest

*Information* measures children’s basic knowledge in regard to a variety of topics, the subtest *Similarities* involves that children display verbal reasoning skills and engage in concept formation. First, raw scores were calculated which were transformed into normalized scores for the respective age group. Since we used two out of three subtests we arrived at the estimated Verbal IQ scores by building a sum of the normalized values, subsequently dividing it by two and finally, multiplying it by three. These steps followed the standard procedure for calculating IQ estimates as proposed in the test manual (Petermann, 2009). In *n* = 7 children the test could not be administered due to concentration issues. See Table 1 for an overview of the measures.

**TABLE 1:** **Overview of measurement points when particular tasks were administered**.

****	**Still-face**	**Social mirroring**	**Mirror self-recognition**	**Delayed self-recognition**	**Verbal IQ**
9 months	X				
12 months		X			
24 months			X		
4 years				X	X

## Results

An overview of descriptive statistics of the study variables is presented in Table 2. In preliminary analyses, in regard to group differences based on gender, girls, on average, were more advanced in their social mirroring skills at 12 months, *t*(67) = 2.25, *p* = 0.03 [girls (*n* = 31): *M* = 1.74, SD = 1.09; boys (*n* = 38): *M* = 1.11, SD = 1.23], and in their MSR at 24 months of age, *χ*^2^(1) = 9.11, *p* = 0.00; *n* = 69, [girls (*n* = 34): recognizers: 85%; non-recognizers: 15%; boys (*n* = 35): recognizers: 51%; non-recognizers: 49%]. Further, girls showed more of a SF smile effect, *χ*^2^(1) = 4.22, *p* = 0.04; *n* = 61, [girls (*n* = 31): SF smile effect: 97% yes, 3% no; boys (*n* = 30): 80% yes, 20% no].

**TABLE 2:** **Descriptive statistics of the study variables**.

****	***N***	%/*M*	**SD**	**Categories/Range**
SF effect mile 9 months	61	0 = 12/1 = 88	0.32	0/1
SF effect gaze 9 months	66	0 = 30/1 = 70	0.43	0/1
SF effect reengagement 9 months	66	0 = 21/1 = 79	0.41	0/1
Social mirroring 12 months	69	1.39	1.20	0–3
Mirror self-recognition 24 months	69	0 = 32/1 = 68	0.47	0/1
Delayed self-recognition 4 years	55	1.80	1.31	0–4
Verbal IQ 4 years	63	107.44	13.72	67–137

mo, months; SF, still-face.

In contrast, boys and girls did not differ significantly in their SF gaze effect [*χ*^2^(1) = 0.00, *p* = 1.00; *n* = 66], nor in their SF reengagement effect, [*χ*^2^(1) = 1.45, *p* = 0.23; *n* = 66]. Further, girls and boys did not differ significantly in regard to verbal IQ, *t*(61) = 0.64, *p* = 0.52, as well as in regard to DSR skills at 50 months of age, *t*(53) = 1.50, *p* = 0.14.

To assess relations among the study variables, correlational analyses were conducted with a two-tailed significance level. As can be seen in Table 3 we found that interindividual differences in social mirroring skills at 12 months of age were predicted by interindividual differences in the SF smile effect at 9 months, while in turn, interindividual differences in social mirroring at 12 months predicted interindividual differences in DSR at 4 years of age. Further, interindividual differences in MSR at 24 months and in DSR at 4 years were related.

**TABLE 3:** **Zero-order point-biserial correlations between the study variables**.

****	**1**	**2**	**3**	**4**	**5**	**6**	**7**
SF effect smile	1						
SF effect gaze	0.12^#^(60)	1					
SF effect reengagement	–0.18^#^(61)	–0.18^#^(66)	1				
Social mirroring	0.29*(47)	–0.11(51)	0.08(51)	1			
Mirror self-recognition	0.15(47)	0.29*^#^(50)	0.01(50)	0.09(51)	1		
Delayed self-recognition	0.01(39)	–0.03(43)	0.01(43)	0.36*^≠^(39)	0.26^+^(49)	1	
Verbal IQ	0.13(45)	–0.05 (50)	0.33*(50)	–0.06^≠^(51)	–0.03(54)	0.22^≠^(48)	1

*p < 0.05; ^+^p < 0.10 (two-sided significance level); ^#^Phi-coefficients; ^≠^Pearson correlation; SF, still face.

To assess the influence of possible mediators (gender and verbal IQ), partial correlations were conducted.

The significant relation between children’s social mirroring skills at 12 months and DSR at 4 years of age *r*_partial_ (36) = 0.32, *p* = 0.048, remained significant when controlling for gender, while the relation between MSR at 24 months and DSR at 4 years of age remained marginally significant when controlling for gender; *r*_partial_ (46) = 0.23, *p* = 0.05, as did the relation between the SF smile effect and social mirroring at 12 months, *r*_partial_ (44) = 0.22, *p* = 0.07.

In order to assess the influence of early social responsiveness on children’s MSR, while also considering possible mediators, in addition to correlational analyses, regression analyses were conducted.

First, a binary-logistic regression analysis (inclusion method) was performed with the complete set of early social responsiveness measures as theoretically important predictors. Further, based on Baron and Kenny (1986) mediation model, we included all possible mediator variables based on if they showed significant or marginally significant correlations with the outcome into the regression analysis. Thus, verbal IQ could be excluded as a mediator variable since it proved to be unrelated to MSR at 24 months (see Table 3), while gender had to be included. The overall model correctly predicted 76.5% of recognizers and non-recognizers. Only the SF gaze effect proved to be an independent predictor of MSR at 24 months (see Table 4).

**TABLE 4:** **Binary-logistic regression predicting MSR at 24 months**.

****	**B**	**Wald**	**Odd’s ratio**	***p***
SF effect smile	0.842	0.363	2.321	0.547
SF effect gaze	2.266*	4.439	9.640	0.035
SF effect reengagement	0.614	0.246	1.847	0.620
Social mirroring	0.193	0.185	1.213	0.667
Gender	1.090	1.249	2.976	0.264
Constant value	–2.502	1.309	0.082	0.253

*p < 0.05; SF, still-face; SF, still-face.

In order to assess the respective importance of early social responsiveness for children’s DSR, while also taking into consideration possible mediators, a linear regression analysis (inclusion method) was conducted with the complete set of early social responsiveness measures as predictors. Based on Baron and Kenny (1986), verbal IQ was included as a possible mediator, while gender could be excluded. As is shown in Table 5, the overall regression model, *F*(6, 32) = 2.32, *p* = 0.056, was marginally significant and explained 30% of variance in children’s DSR at 4 years of age. Looking at single predictors, interindividual differences in social mirroring skills at 12 months were found to significantly predict DSR at 4 years of age, beyond verbal IQ at 4 years as another significant predictor and MSR at 24 months as a marginally significant predictor.

**TABLE 5:** **Regression analysis to predict delayed self-recognition at 4 years of age**.

****	**ß**	***T***	***R*^2^**	***F*-value**
SF effect smile	–0.24	–1.43		
SF effect gaze	–0.06	–0.35		
SF effect reengagement	–0.19	–1.16		
Social mirroring	0.43*	2.69		
Mirror self-recognition	0.29^^^	1.84		
Verbal IQ	0.34*	2.12		
			0.303	2.32^^^

^^^p < 0.10; *p < 0.05; SF, still-face.

## Discussion

This longitudinal study had two major aims: It explored the conceptual coherence of the self as a construct, as well as the specificity of the social origins of the self. It included precursor abilities and measures of self-awareness from infancy through toddlerhood to preschool age, as well as control measures. Most importantly, the findings suggest quite specific developmental pathways. Using (cf., Rochat’s, 2003) terminology, one such pathway appears to lead from infants’ gaze reaction to contingency disruption at 9 months to self-“identification” at 24 months, while the other leads from self-awareness in a social imitation game at 12 months to self-“permanency” at 4 years of age. These developmental pathways indicate a fairly long-term continuity of social cognition in regard to self-understanding that is consistent with the long-term continuity of social cognition in regard to other-understanding as indicated by developmental pathways from infants’ understanding of goals and intentions to their later theory of mind (cf., Aschersleben et al., 2008; Wellman et al., 2008; Thoermer et al., 2012). Further, interindividual differences in regard to the identified self and in regard to the permanent self proved to be moderately related, independently of gender or verbal IQ. Thus, the self seems to develop as a multi-dimensional, and at the same time, (moderately) coherent concept.

The findings provided some support for theories emphasizing the role of social interaction in the development of a concept of self (e.g., Moore, 2007; Rochat, 2009). Specifically, interindividual differences in children’s responses to contingency disruption at 9 months as indicated by their gaze predicted MSR, while sensitivity toward social mirroring predicted DSR. This is consistent with Rochat’s (2009) idea that children do not only use reflecting surfaces of all kinds, but their social world as a mirror. Thus, infants expect others to project their inner self back at them, just like a real mirror would do. Note that this expectation might have been rapidly formed during infants’ early dyadic social interactions with their caregivers and might not be entirely endogenous, since exogenous, parental factors have been shown to impact children’s behavior during SF (e.g., Rosenblum et al., 2002).

Further the results are consistent with cross-cultural findings (e.f., Keller et al., 2005) showing that toddlers show earlier MSR-skills if they are socialized in cultures with a distal parenting style promoting attentiveness toward the human face. Note also that the correlational link between gaze behavior during the SF task and MSR found in this study supports theories proposing that the development of domain-specific cognitive structures supports the development of MSR-skills (e.g., Neisser, 1995). More specifically, it was children’s sensitivity toward interruption as indicated by gaze which predicted mark removal, while the arguably more emotional-evaluative reactions during the SF task, such as smiling and trying to re-engage the partner, were not related to mark removal. It is possible that smiling and re-engagement would be related to more emotional-evaluative reactions to the mirror image (such as puzzlement or coy reactions). This is a task for future research, since those behaviors are expected to develop well before mark removal. For instance, Reddy (2003) proposes an affective-engagement account claiming that the experience of self as an object to others as reflected in affective responses or coy reactions (i.e., smile with gaze and/or head aversion), occurs at a very early age, around 2 months. Links between the SF situation and these measures in the mirror situation need to be explored in future research involving younger age groups.

Again in favor of specific rather than more general developmental links and in favor of the self being a multi-dimensional construct, our data showed that while predicting DSR, social mirroring in infancy did not predict MSR. Consistent with our findings, a study in 9-to-18-month olds by Agnetta and Rochat (2004), using a modification of Meltzoff’s social mirroring task, also did not find significant predictive relations between social mirroring and MSR. Thus, while these skills may emerge around the same age (Nielsen and Dissanayake, 2004) and while interindividual differences may be concurrently related, especially later in development (Asendorpf and Baudonniére, 1993), social mirroring skills toward the end of the first year of life do not seem to be predictive of MSR. However, they seem to predict DSR. There are several possible explanations for these differential relations.

Nielsen and Dissanayake (2004) explained the missing link between MSR and synchronic imitation skills found in their own research referring to Perner’s (1991) developmental theory. They argue that while secondary representations can be applied in one field of development (e.g., featural information), children might not be able to apply them in another field of development (e.g., temporal information). This interpretation implies domain-specific, rather than domain-general pathways of self-development and is thus empirically supported by the results of this study showing very specific relations. During the SF task, as well as during the mirror rouge test children have to use featural information to detect contingency. Consistently, the MSR and the SF gaze effect in this study were also related. In contrast, as is argued by Meltzoff (1990) in social mirroring contingency detection based on the infant’s understanding that an interaction partner behaves “like me” rather than on featural information as in MSR is assessed. In DSR, children have to use featural, as well as temporal cues to represent a temporally-extended self. Consistent with this interpretation, both MSR and social mirroring predicted DSR. Further, another explanation would be that social imitation gets increasingly complex and with increasing age it is supposed to signify a higher level of mental state understanding and higher meta-cognitive skills. More specifically, while the early detection of “being imitated” in Meltzoff’s task may be based on mere surface detection of temporal contingency with increasing age it might reflect infants’ intention understanding (Tomasello, 1995; Striano and Rochat, 1999). Thus, the infant, who understands that the interaction partner systematically matches his intended acts, will thereupon attribute the *intention* to behave “like me” to the imitating partner. Similarly, Agnetta and Rochat (2004) argued that while at 9 months the discrimination of an imitating adult is based primarily on the detection of contingency and a sense of self-agency, toward the end of the first of year of life it is based on intention understanding. Thus, social mirroring at 12 months is likely to already reflect higher cognitive processes. Consistent with this interpretation, social mirroring skills predicted DSR as the cognitively more demanding measure of self-awareness.

While experimental research needs to identify the underlying cognitive mechanisms of the developmental links identified in this study, it seems plausible that children’s underlying metacognitive awareness of being intentionally imitated as is the case in the social mirroring task at 12 months and their metacognitive awareness of having experienced what can be seen on the video at 4 years of age, may be the source for the developmental link between the tasks. Longitudinal research, using metacognition as an outcome measure, could shed light on that interpretation. Finally, while the present longitudinal study only started at the age of 9 months, theories of self-development have viewed the first few months of life as particularly important. For instance, children’s awareness of their own body as an object and its relations to other objects has been conceptualized as a unique part of self-awareness (e.g., Brownell et al., 2007; Moore et al., 2007). Rochat (2009) proposed that it is from the second month of age that infants engage in reciprocal exchanges and thereby begin to objectify themselves as objects of shared attention. Already at the age of 6 weeks, according to Rochat (2003), the “situated self” is established, a sense of how one’s own body is situated in relation to other entities in the environment. Therefore, to further complement the picture, future longitudinal research on developmental precursors of MSR and DSR should include measures of social responsiveness in the first months of life.

The present longitudinal study supports the notion that MSR seems to develop earlier than the permanent self which involves the understanding that the self is invariant over time (Povinelli et al., 1996; Suddendorf, 1999). Importantly, since identification of the mirror image is tied to the temporal simultaneity of the body and its mirror reflection, future research needs to shed light on the exact nature of the capacities underlying the ability to recognize one’s own mirror image. Interestingly, while related to different precursor abilities, MSR and DSR were found to be moderately developmentally related to each other, indicating conceptual continuity. This result is in support of Rochat (2003) theory of self-development. Rochat compares the self to an onion with different layers. Thus, the self as a whole always comprises earlier and later-developing stages. More specifically, one possible developmental mechanism linking MSR and DSR proposed by Gallup (1998; cited in Nielsen and Dissanayake, 2004) is that during MSR children need to engage in introspection in order to focus on themselves and to become the object of their own attention. Thus, self-recognizers possess higher introspection skills than non-recognizers. Subsequently, if applying introspection across a variety of contexts during development, combined with meta-representational abilities, introspection should not only promote children’s time-restricted self, but also their temporally extended sense of self.

A limitation of this study is that it did not include a socially or culturally diverse sample. Cross-cultural research comparing cultures with distal and proximal parenting styles, as well as research in clinical samples need to corroborate the developmental links identified in this study. Are deficits in social responsiveness and imitation skills (e.g., in autistic children) related to decreased MSR- and DSR-skills?

In sum, the present longitudinal findings show that infants make use of their social world to form an understanding of who they are. This seems to result in very specific, rather than general developmental links between early social responsiveness and children’s later understanding of self. To further explore these specific developmental pathways more longitudinal work, focusing on interindividual differences, is clearly needed. Note that the same developmental links identified in self-development might also be found when studying children’s early understanding of others (cf., Moore and Corkum, 1994). Similarly, on the neural level, there seems to be a shared, while not completely overlapping, representation network for self and other (Decety and Sommerville, 2003). Thus, future studies should look simultaneously at the development of self and other. In sum, the present findings suggest that one of the multi-faceted interrelations between self-understanding and other-understanding originates from infants’ understanding of the other’s intention to “act like me” which seems to lay the ground for an advanced concept of the temporally extended self.

### Conflict of Interest Statement

The authors declare that the research was conducted in the absence of any commercial or financial relationships that could be construed as a potential conflict of interest.
